# Preliminary development and evaluation of a mechanical handwriting assistive device to support individuals with movement disorders

**DOI:** 10.3389/fresc.2024.1418534

**Published:** 2025-01-17

**Authors:** Gabrielle Lemire, Thierry Laliberté, Katia Turcot, Véronique H. Flamand, Alexandre Campeau-Lecours

**Affiliations:** ^1^ Department of Mechanical Engineering, Université Laval, Quebec, QC, Canada; ^2^ Centre for Interdisciplinary Research in Rehabilitation and Social Integration, CIUSSS de la Capitale-Nationale, Quebec, QC, Canada; ^3^ Department of Kinesiology, Université Laval, Quebec, QC, Canada; ^4^ School of Rehabilitation, Université Laval, Quebec, QC, Canada

**Keywords:** assistive technology, cognitive rehabilitation, handwriting, movement disorder, orthotic device, self-help devices

## Abstract

Individuals with movement disorders often face challenges in writing independently due to factors such as spasticity, lack of precise motor control, muscle weakness, and tremors. This paper aims to develop a handwriting assistive device (HAD) for individuals with movement disorders, to stabilize the motion of user's hand, through initial needs assessment, iterative design, and a preliminary evaluation. The research is scoped to include only initial testing with a small user group, six potential users with movement disorders, providing foundational insights for future refinement. The findings from the initial needs assessment revealed that current assistive technologies do not fully meet handwriting challenges for individuals with motor impairments. The HAD prototype, developed with adjustable damping mechanism and customizable handles to suit different levels of motor control, enabled steadier handwriting in preliminary testing with six participants. Children drew shapes more accurately, and some traced letters they couldn't otherwise. The adult participant showed greater fluidity and legibility, completing tasks 4.81 times faster with the HAD. The qualitative feedback indicated the device's potential to enhance handwriting independence and usability across age groups. Future prospects for this study include developing an electronic version of the HAD, allowing real-time adjustable damping to better support users' voluntary movements while further stabilizing involuntary motions.

## 1 Introduction

Movement disorders refer to a group of neurological conditions characterized by abnormal motor function, affecting both voluntary and involuntary movements. These disorders can be present from birth or develop later in life and can vary significantly in terms of severity and impact. In children, movement disorders may be associated with conditions such as cerebral palsy, dystonia, and developmental coordination disorder (DCD), where the motor control and coordination necessary for daily activities, including handwriting, are impaired. As children grow, motor deficits can interfere with their ability to perform age-appropriate tasks, hindering academic performance and social participation. In adults, movement disorders like Parkinson's disease, multiple sclerosis, or stroke-related sequelae can lead to tremors, rigidity, bradykinesia (slowness of movement), and other symptoms that disrupt fine motor skills, including handwriting. These conditions may result in illegible writing, difficulty maintaining consistent letter formation, and slower task completion. Both children and adults with movement disorders often face challenges in achieving independence in tasks like handwriting, which can significantly affect their self-esteem and quality of life.

Occupational therapists use a variety of approaches to assist these children with motor impairments, which include recommendations of various technologies (for example, keyboard-based strategies), especially when children cannot keep up with the volume of classroom work (1). However, it is recognized that there are important differences in the learning process of writing between typing on a computer and writing by hand. Indeed, using a keyboard and handwriting develop different parts of the brain (2). Even though using a keyboard is less demanding for children, relying on digital devices can hinder learning processes (3). Moreover, traditional handwriting has many advantages for the development of children. It helps memorization (4), letter recognition (3), and spelling (5). Note-taking is also more efficient when it is handwritten instead of being typed (6) because it helps memorize what was written. It is also reported that math problems can be solved more easily when the approach is written down (7). Recent studies have shown that handwriting can enhance the generation of ideas, as well as cognitive and information processing abilities, more effectively than typing on digital devices (3, 8). Children have more ease in learning letters when they write them down instead of simply seeing them (9). Therefore, uncoordinated or involuntary movements that lead to handwriting difficulties can have serious consequences on the academic, social, and emotional development of children (10).

Existing studies have proposed some designs to help people living with movement disorders write. An assistive drawing device was developed to help children with cerebral palsy draw, but the device had a small range of motion and was developed for specific users with cerebral palsy (11). Certain designs with bigger pencil grips and ergonomic pens^1^ may help children who are not capable of handling small-diameter pens. For people with Parkinson's disease, anti-tremor gloves^2^ may help with tremors. A haptic device (12) and the Clinical Kinematic Assessment Tool (13) can help children learn the movement for writing words and sentences. All the assistive technologies (AT) help children communicate and socialize (14), but none of them help with spasms. While a variety of solutions have been proposed, the literature also points to a number of factors that limit user adoption of AT devices in general, including high cost, difficulty of operating devices, deceiving performance, and insufficient adaptation to the user's needs (15, 16). From informal discussions with occupational therapists in our rehabilitation institute (Quebec, Canada), it was revealed that many people living with movement disorders cannot write by themselves (or write with difficulty) and that in many cases, no existing AT was able to fully meet their needs and help them with handwriting.

In that context, the overall goal was to develop and evaluate a handwriting assistive device (HAD) to help children living with movement disorders associated with handwriting and drawing. To restrict the scope of the article, we first aimed to address two types of motor disorders: (a) contractures due to spasticity (in the upper limbs) or joint deformities (which prevent the user from holding the pencil correctly), and (b) abrupt movements (e.g., spasms, ataxia, dystonia). The specific objectives were threefold: (1) to establish the current situation related to handwriting amongst the target population; (2) to design an HAD prototype that would stabilize the user's motion and enable handwriting and; (3) to perform a preliminary evaluation of the HAD prototype to assess its performance and to guide the development of future iterations.

## 2 Objective 1 - current situation and needs assessment: methods

### 2.1 Initial stakeholder input

A round table discussion of six occupational therapists from CIUSSS Capitale-Nationale and two engineers from the research team was conducted to discuss the current situation and needs of children related to handwriting. Occupational therapists included in the study had a minimum of two years of clinical experience specifically working with children with movement disorders, with participants' clinical experience ranging from 2 to 35 years. The roundtable aimed to gather expert opinions and identify the key features required for a new handwriting assistance device (HAD) that could effectively support handwriting tasks for children aged 7–12. The children included in the study were experiencing various movement disorders impacting handwriting, such as cerebral palsy, developmental coordination disorder, and tremor-related conditions (see Table 1). The studies involving human participants were reviewed and approved by CIUSSS Capitale-Nationale ethics committee, # 2018-427, RIS_2017-579, CIUSSS-CN. The studies were conducted in accordance with the local legislation and institutional requirements. The participants provided their written informed consent to participate in this study and written informed consent for participation in this study was provided either by the participants or their parents/legal guardians.

**Table 1:** Summary of participant characteristics.

Characteristic	Details
Participants	6 participants (5 pediatric, 1 adult)
Age range of participants	7–12 years (pediatric group); 45 years (adult)
Primary movement disorders	Language impairments and mild motor difficulties (pediatric group); Cerebral Palsy (adult)
Motor difficulties	Mild to moderate motor difficulties in pediatric group; Spasticity and uncoordinated movements in adult
Stakeholder sample (Therapists + Engineers)	6 occupational therapists with 2+ years of clinical experience and 2 engineers from the research team

### 2.2 Questionnaire and technology review

A questionnaire was answered by occupational therapists to evaluate how current HADs address the needs of children and to assess the potential benefits of a new device. The questionnaire gathered insights on whether existing devices were meeting user needs and how a new solution could positively impact children's development and self-esteem. While this questionnaire was not a standardized, validated tool, it was developed based on common metrics in handwriting assistance literature to capture specific needs and therapist feedback. Similar validated questionnaires exist in the field, such as the Canadian Occupational Performance Measure (COPM), but this custom questionnaire was used to gather targeted insights specific to this prototype development process.

Following the questionnaire, a technology review (images, videos, demonstrations of available systems) was presented to the group for open discussion in order to examine the perceived advantages and drawbacks of each solution, based on the users' experience with such devices and the users' insights based on practice. This milestone allowed a better understanding of the important features and constraints that the new HAD would need to address. To finalize the data collection, a semi-structured group interview was performed to collect any remaining pertinent information. Written notes were taken by the research team during this interview.

## 3 Objective 2: development of the HAD

### 3.1 General overview

**Figure 1:**
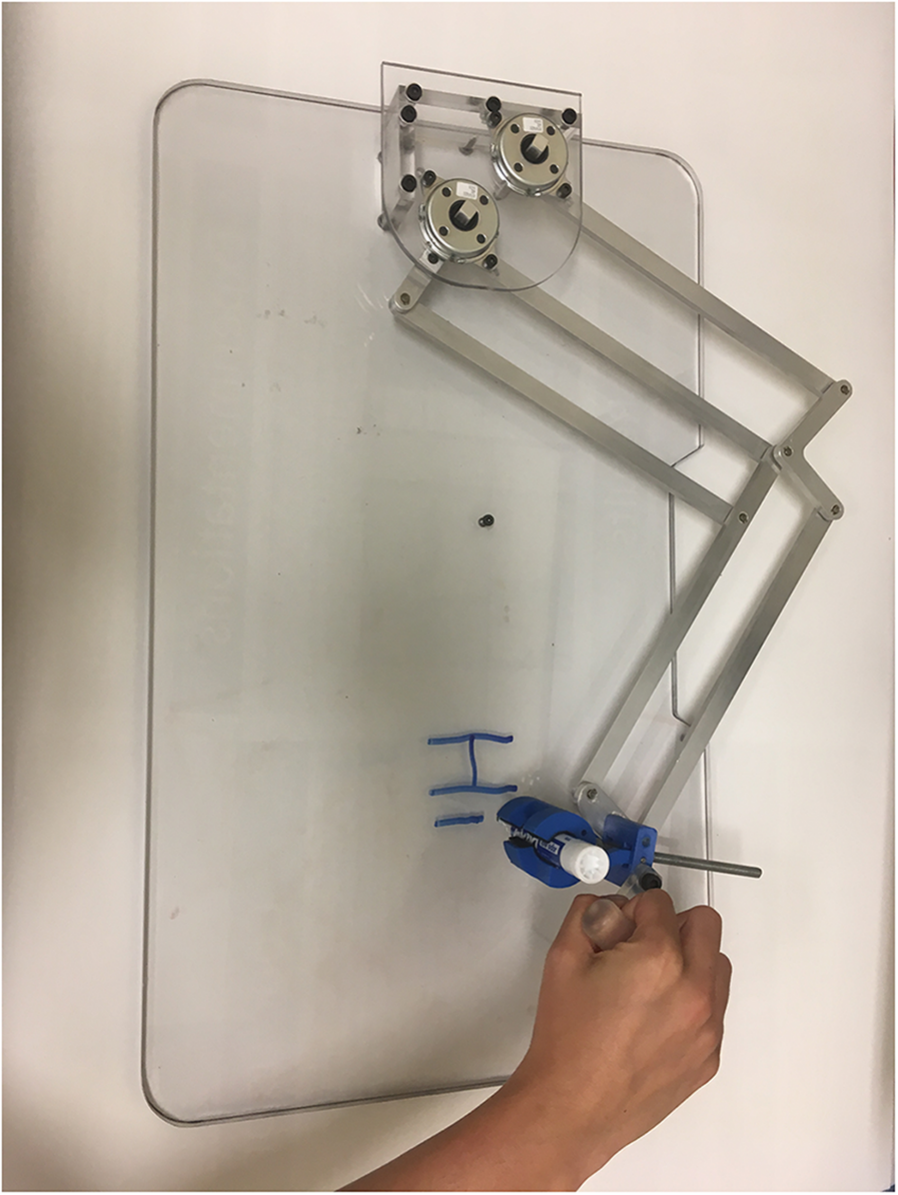
HAD prototype.

During discussions at the roundtable, different solutions were explored and an HAD prototype was developed via an iterative process in collaboration with occupational therapists and researchers in engineering and rehabilitation, through a user-centered approach based on Design Thinking (a process that places the individual and his/her needs to the center of the reflection and allows him/her to participate actively in the innovation) (17). The main characteristics that were aimed at in the design were to automatically maintain the pen in a constant orientation and to reduce the impact of uncoordinated movements. The final proposed system is shown in Figure 1.

### 3.2 Mechanism

This section presents the mechanical design of the HAD. The design was made of a two-degrees-of-freedom (2-DOF) 5-bar mechanism. It includes a parallelogram that allows controlling the second rotation from the base. It is desired to constrain the rotation of the end effector. To do so, two parallelograms were added; one for each bar. This created a HAD where the pen is always in the same orientation with respect to the user, and the control of the two DOFs is at the base of the mechanism. More details on the mechanism development are provided in the article by Lemire (18). As mentioned, the HAD should be compact and should cover an entire letter format sheet, either in portrait or landscape orientation.

### 3.3 Pen holder and handles

The pen is held in place with a pen holder mechanism. It is designed so that it can be used with pens of many sizes, with diameters from 8 mm to 20 mm. A spring allowed the pen holder to automatically open when the pressure is released from the tightening screw, as seen behind the pencil in Figure 1. The pen holder design is relatively small so it is possible for users to hold the pen directly if they display the motor skills required to hold a pen.

**Figure 2:**
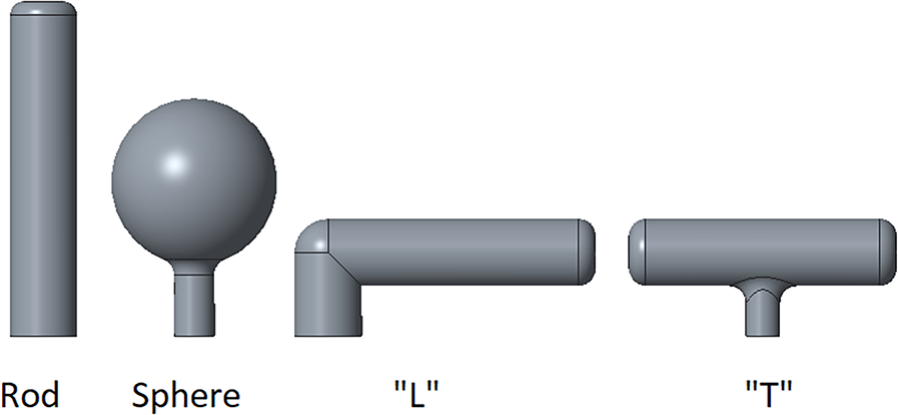
The four available handles to control the mechanism.

Since the users' capacities are very limited, it is important for the HAD to be adaptable. To this end, handles of different shapes, orientations, and sizes can be added on the side of the pen holder. Four handles are designed. The available shapes are a “T”, an “L”, a sphere, and a rod, as seen in Figure 2. They are available in small and large sizes. It is also possible to easily design new ones for more customizability. Depending on the user's preferences, the handle can be fixed in a certain orientation from the base or it can rotate around its axis. All the adjustments on the pen holder mechanism and the handle can be performed in a quick and easy manner so that many users can use the same device.

### 3.4 Stabilizing the movements

Since target users might make uncoordinated movements, an important part of the design objective is to assist the movements in order to limit the effects of such movements. To this end, rotary dampers are added to the HAD joints. As a result, the dampers damp the rotary motions of the mechanism's bar. As these dampers are linked directly to the bars that control the two DOFs, they move proportionally to each DOF. For a given Cartesian displacement of the end effector, the resulting motion at the joints will vary depending on the end effector's position. The dampers will thus produce different Cartesian damping values for different positions of the end effector in the workspace. This might create an unintuitive feeling for the user at the end effector since: (1) for a given position of the end effector, the Cartesian damping could be different depending on the movement direction (e.g., the strong damping ratio in a given Cartesian direction (e.g., forward-backward) with a low damping ratio in another orthogonal direction (e.g., left-right); (2) for a given movement direction, the intensity of the damping could be different for various end-effector positions). An optimization was thus performed in order to obtain a Cartesian damping ratio as uniform as possible on the mechanism's workspace. To this end, it was first required to characterize the effect of the damping transformation from the angular movement to the Cartesian linear movement. This has allowed us to determine the required mechanism dimensions (i.e., bar length) and characterize where the damping is mostly uniform in both directions.

### 3.5 Matrix conditioning

The HAD was first characterized using the Jacobian matrix. The Jacobian matrix of the HAD reflects the relation between the Cartesian velocity of the end effector and the angular velocity of each joint. This matrix is thus useful to characterize the kinematics of the HAD and is represented in Equation 1.

(1) $$\begin{array}{c} J=\left[\begin{array}{cc} -l_{1}\sin\;(\theta_{1}) & -l_{2}\sin\;(\theta_{2})\\ l_{1}\cos\;(\theta_{1}) & l_{2}\cos\;(\theta_{2}) \end{array}\right] \end{array}$$

**Figure 3:**
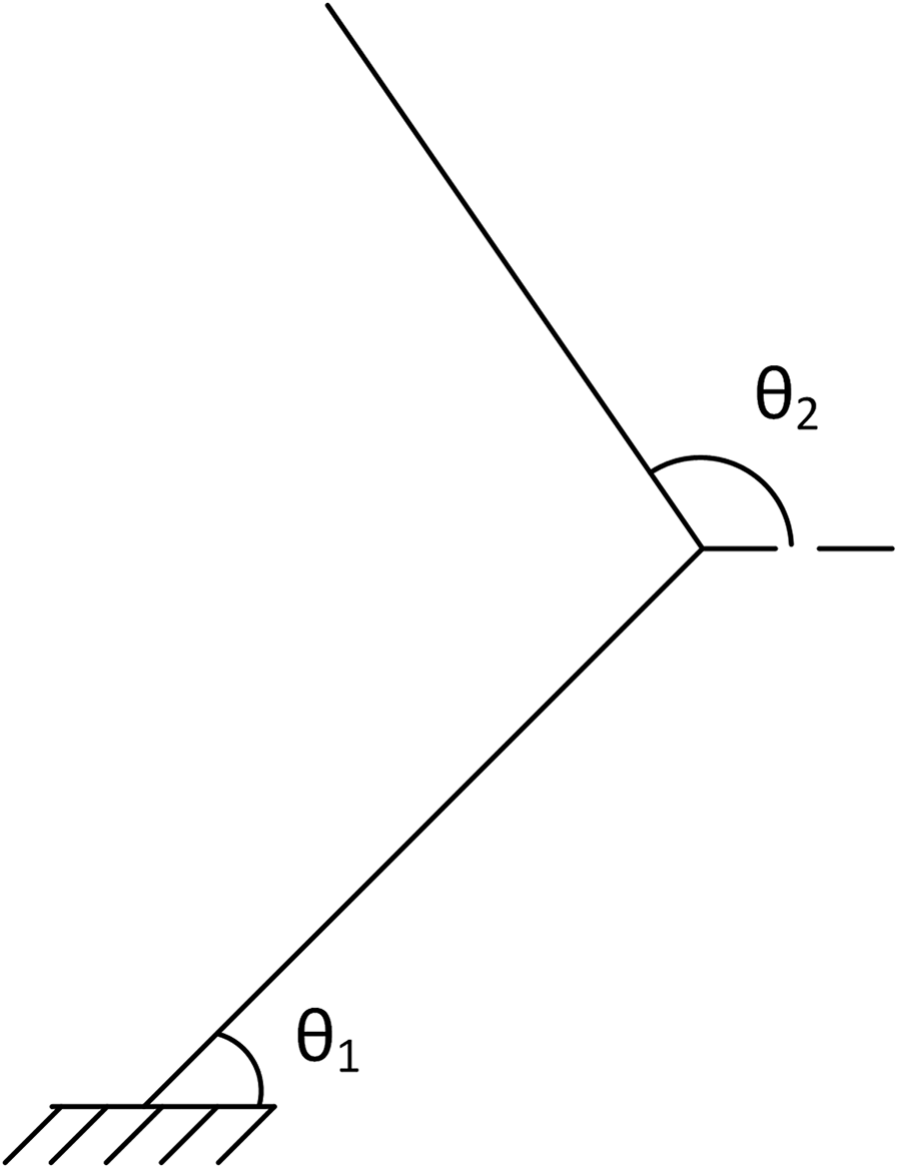
Angle definition for jacobean.

**Figure 4:**
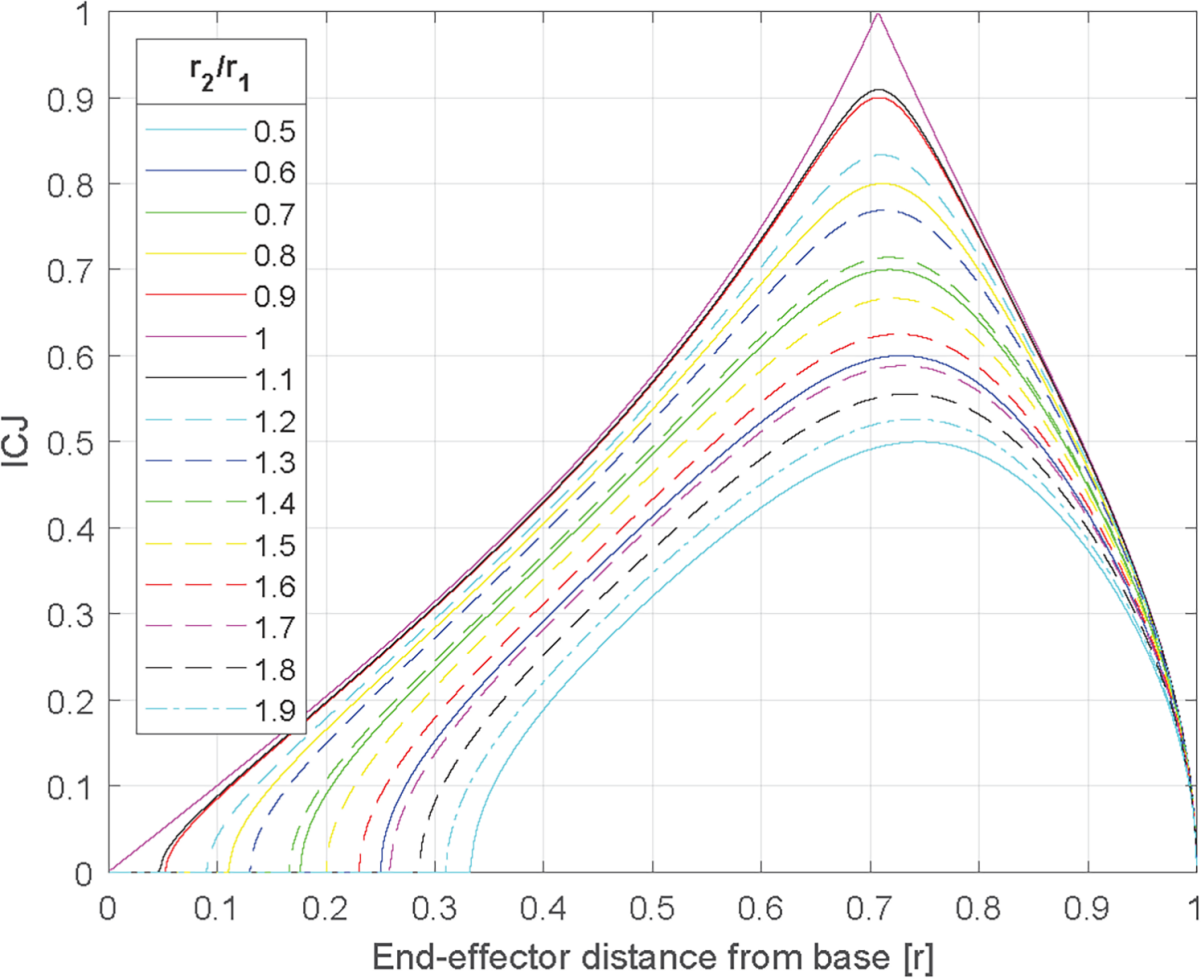
Inverse of the conditioning depending on the length ratio of the two bars.

The angles are defined in Figure 3. With the inverse of the conditioning of the Jacobian (ICJ), it is possible to characterize the maximal relative error between angular and Cartesian displacements of the HAD in its working space since it gives indications of the dexterity of the HAD in general. Equation 2 presented the conditioning of the Jacobian while Equation 3 presented the ICJ. The results of the ICJ are between 0 and 1. The closer to 1, the more uniformly the HAD reacts (damping in a given Cartesian direction is similar to damping in another direction). It can be shown that for given link lengths, the ICJ depends on the distance between the base and the end effector's position. In addition, for given link lengths, the absolute values of the link lengths do not influence the conditioning, but the relative ratio (*l*_2_/*l*_1_) does. Figure 4 shows the results for the HAD with different possible normalized lengths of bars. The *y*-axis represents the ICJ. The *x*-axis represents the distance r from the base using normalized link lengths (*l*_2_/*l*_1_ = *r*) for a position of “*r* = 0” (HAD at the base) to “*r* = 1” (HAD fully extended). Figure 4 also shows different curves with different link length ratios (*l*_2_/*l*_1_). The HAD inverse of the dexterity is null at these two positions (*r* = 0 and *r* = 1) for all link length ratios since these positions are singularities. The point at the highest inverse of the maximal relative error is at a distance of approximately $\frac{\sqrt{2}}{2}*\;(l_{1}+l_{2})$ for all link length ratios. Ideally, the HAD should lead to curves with values closer to 1 and high values throughout the workspace. The best curve is the one with an equal link length (*l*_2_/*l*_1_ = *r*). As the ratio between the two link lengths gets further from 1, the inverse of the maximal relative error decreases. For instance, this means that for a ratio of 0.5 for a given position of the end effector, the Cartesian damping in a given direction is far from equal than in another direction. An equal link length was thus chosen for the prototype.


(2) $$\begin{array}{c} \kappa (J)=\|J^{-1}\|\|J\| \end{array}$$

(3) $$\begin{array}{c} ICJ=\frac{1}{\kappa (J)}=\frac{1}{\left\|J^{-1}\|\|J\right\|}\; \end{array}$$

### 3.6 Effect of torque at each joint

**Figure 5:**
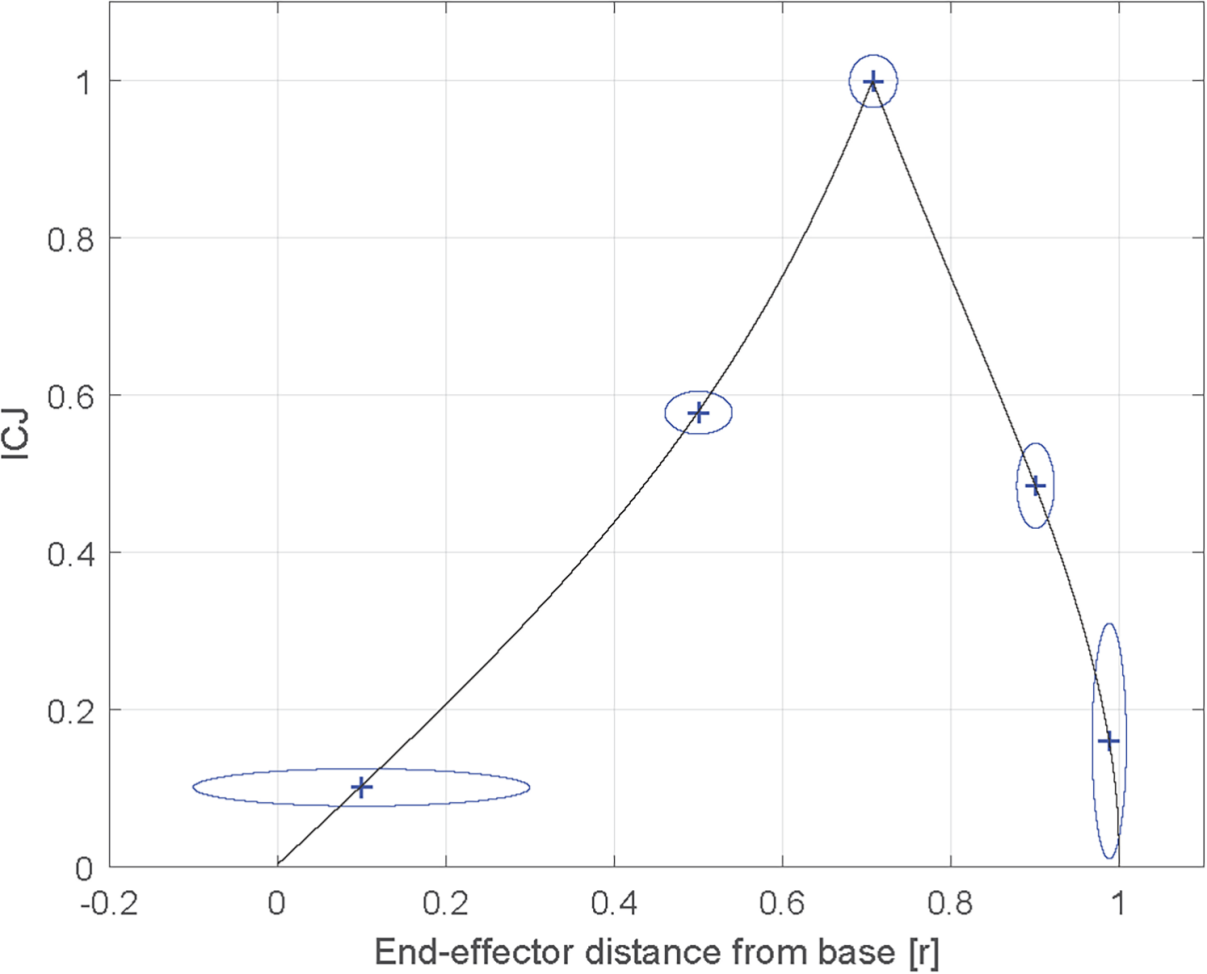
Inverse of the conditioning with the results of the force values at the end effector with specified torque values at the base.

While Figure 4 shows the ICJ for different positions of the end effector, it does not provide tangible information about the resulting uniformity of the Cartesian damping, which would prove important in choosing the HAD's workspace. To this end, simulated torque values ranging from −1–1 Nm acting on both joints ($J_{1},\;\;J_{2}$) have been applied and thanks to the Jacobian matrix, the Cartesian force values at the end effector were found. Figure 5 shows the results of the computation added to Figure 4 curve with equal length (*l*_2_/*l*_1_ = 1) to represent how the Cartesian force values (in the horizontal and vertical axes) differ. The force values are equal when the ICJ is equal to 1, and tend to differ as the ICJ gets smaller. For instance, at *r* = 0.5the vertical Cartesian force is 0.058 N and the horizontal force is 0.01 N, while at *r* = 0.707, both the vertical and horizontal forces are 0.0071 N. For a point even farther from *r* = 0.707, such as *r* = 0.1, the vertical force is 0.005 N and the horizontal force is 0.05 N.

### 3.7 Effects of angular damping on linear movements

After performing the analysis of the ICJ and the force values at the end effector depending on the torque values at the two DOFs, it would be interesting to find the reflecting Cartesian damping in the working plane. As demonstrated in the previous subsection, the force values detected in the two Cartesian directions at the end effector are not equal if the ICJ differs from 1. The Cartesian damping felt by the user is characterized by the linear damping matrix which is obtained with the Jacobian and angular damping matrices as shown in Equation 4.

(4) $$\begin{array}{c} D_{l}=J^{-t}D_{a}J^{-1} \end{array}$$

**Figure 6:**
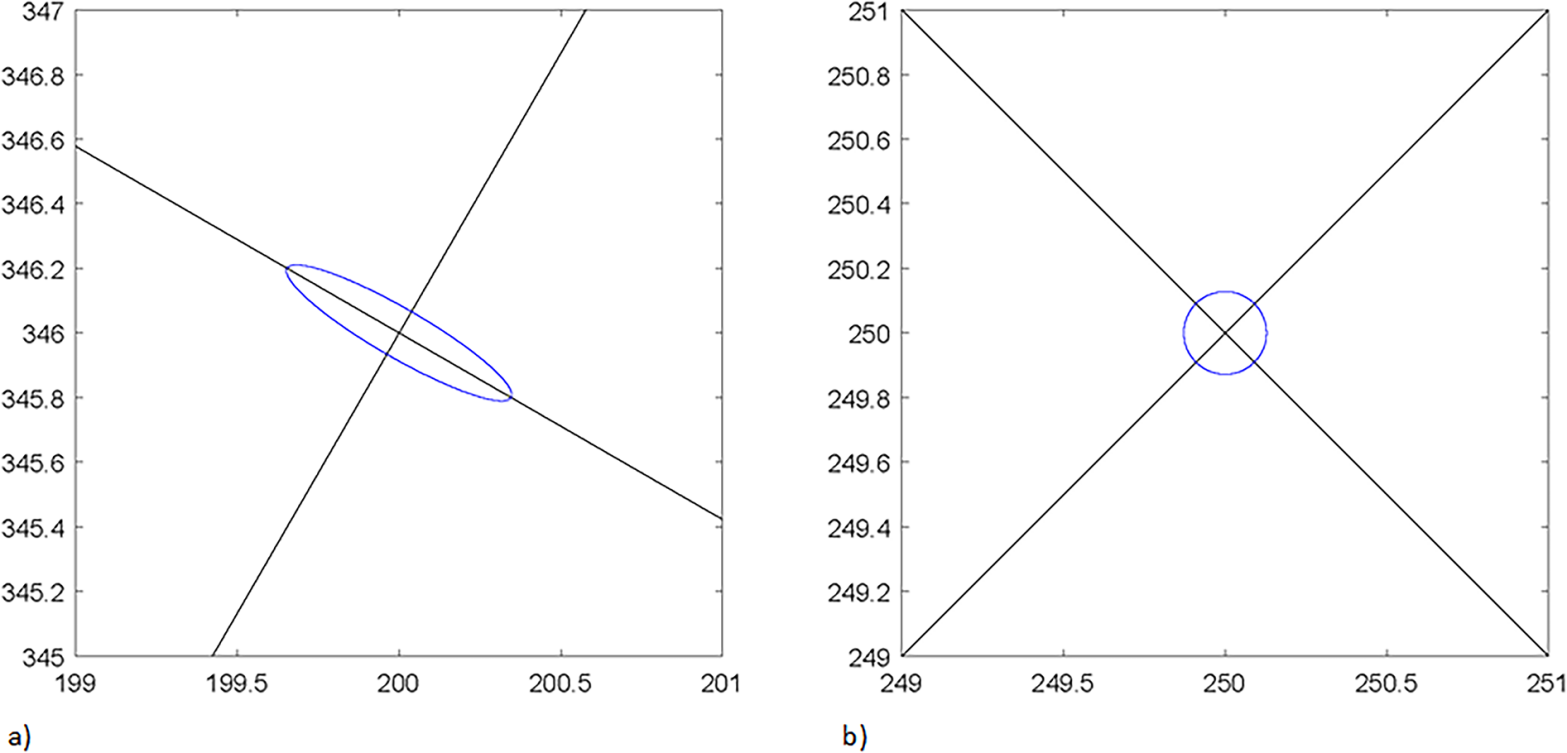
Ellipses of the eigen values and lines of the eigen vector; **(a)** at point (200; 346) **(b)** at point (250; 250).

The results of the reflecting linear damping highlight important information, namely that there are two Eigen vectors representing the main axes of the damping and that these axes are orthogonal with each other as shown in black in Figure 6. The two parts of the figure represent different points in the workspace. The left one is from a point at (200, 346) mm from the base, and the right one is from a point at (250, 250) mm from the base, which represents the best position of the HAD. For equal values of damping coefficients, one Eigen vector passes through the origin of the working space, and the other one is perpendicular to that vector.

Another important information is the Eigen values which indicate the ratio between the minimal and maximal Cartesian damping for a given position of the end effector. They are shown in blue in Figure 6. This information is similar to the one in the preceding section, but with another metric (the damping instead of the force) which presents complementary information to take into account in the design.

The design of the mechanism is then faced with a compromise. It is first desired to obtain a mechanism where the damping feels as uniform as possible over the workspace of an 8.5″ × 11″ sheet of paper. However, it is also desired to limit the overall dimension of the mechanism so the HAD is efficient but not too cumbersome. Based on the experiments and the theoretical work that was presented, it was determined that the best compromise was to set the bar length to have a minimum ICJ of 0.16 all along the workspace, which would lead to two bars of 250 mm each. Having a higher value of minimum ICJ creates a much larger HAD.

## 4 Objective 3: preliminary evaluation

### 4.1 Participants and recruitment

Six participants completed the evaluation. Participants are included if they had motor difficulties and had trouble handwriting on their own. The first five participants were children (age 7–12) from a school in the CIUSSS Capitale-Nationale (Québec, Canada) living with language impairments and light motor difficulties. The sixth participant was an adult (45 years old) living with cerebral palsy and was not able to write on his own due to spasticity and uncoordinated movements. The project was approved by the local ethics committee (Ethics # 2018-427, RIS_2017-579, CIUSSS-CN) and informed consent was obtained from each participant.

### 4.2 Experimental procedure

Each person participated in an individual videotaped testing session, which lasted approximately 30 min. Participants first had time to familiarize themselves with the HAD until they felt ready and confident (approximately 2–3 min), and then the experiment with the HAD started. Users were asked to draw simple forms with their dominant hand with their usual pen (without the HAD), and then with the HAD. The shapes were, in order of increased difficulty for handwriting:

1. Vertical line
2. Horizontal line
3. Circle
4. Vertical cross
5. Oblique
6. Cross
7. Triangle
8. Name or nickname of the participant.

The forms were executed until the participant reached his/her limit.

### 4.3 Performance of variables

At the beginning of each session, sociodemographic and clinical data were collected using a homemade questionnaire on age, gender, and diagnosis. Then, during the evaluation without and with the HAD prototype, qualitative analysis was conducted for the first five participants and the task completion time was measured (for each shape independently) along with a measure of the general writing quality for the last participant.

## 5 Study results

### 5.1 Results: objective 1

**Figure 7:**
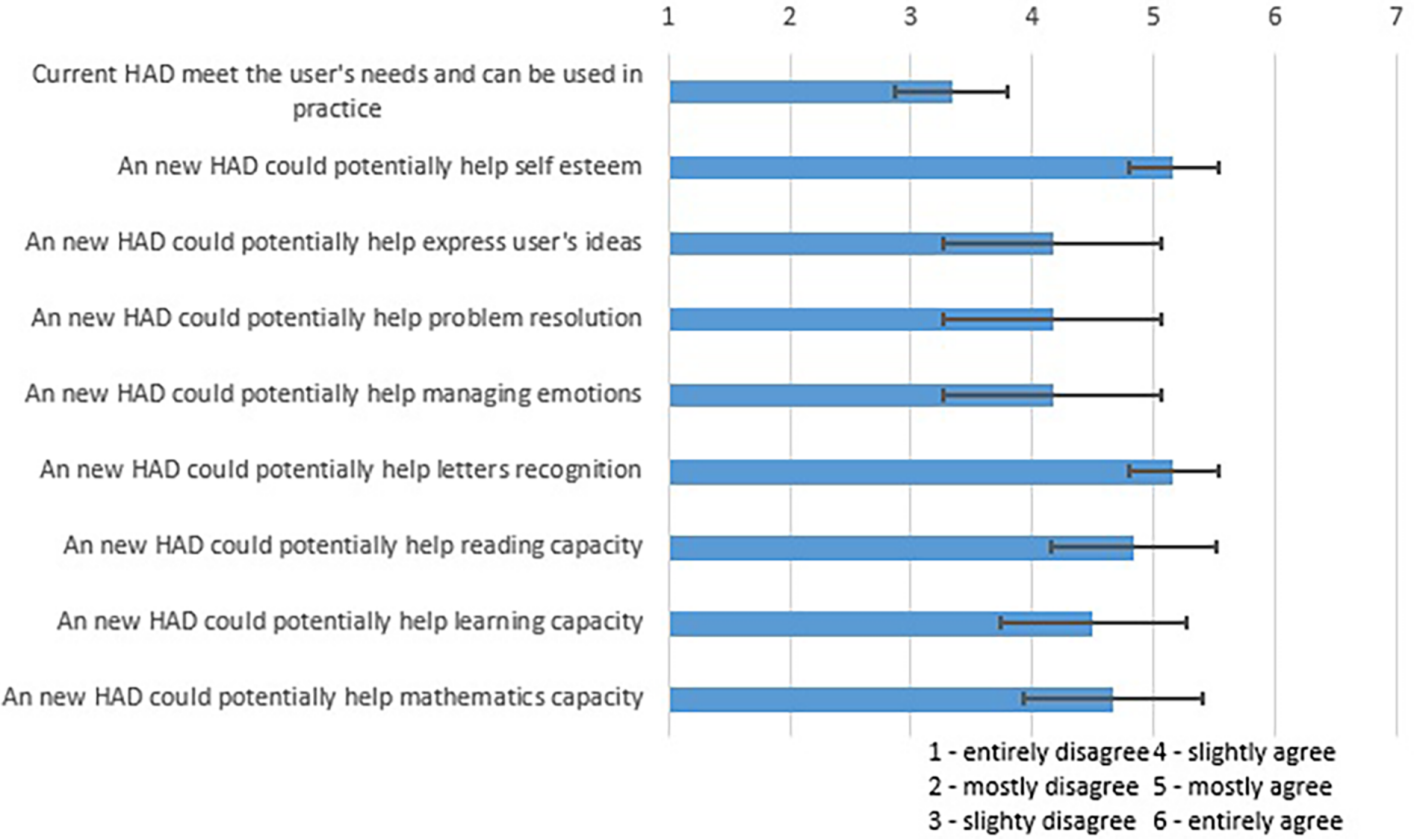
Results of a roundtable with occupational therapists on the possible efficiency of a handwriting device.

The questionnaire's results are shown in Figure 7 and present the mean and the variance for each question. Results from this questionnaire showed that, in the opinion of the participants, the current HADs did not meet the user's needs and could not be used in practice. The figure also shows that, according to them, a new HAD could be beneficial for potential users in many facets of their lives. The occupational therapists also thought that the proposed device would be easily accepted by the parents of potential users as it would help with the handwriting of their children.

A clearer understanding of the advantages and drawbacks of different existing systems emerged from the focus group discussion. When asked about current available HADs, occupational therapists said that they had seen and worked with some such as heavy pens, anti-tremor gloves, larger pencil grips, and the Blackburn orthosis. It was revealed that the current ATs do not help children living with contractures due to spasticity or joint deformities and spasms. The final HAD should be compact and should cover an entire letter format sheet, either in portrait or landscape orientation. This feature would ensure better functionality for children with severe motor impairments.

### 5.2 Results: objective 2

The results from user testing and performance evaluations (Figure 1) demonstrated that the Handwriting Assistance Device (HAD) effectively supported users with motor impairments by improving pen orientation and control. Occupational therapists noted its potential to help children with severe motor impairments, particularly with its compact design and adjustable handles. Focus group discussions highlighted the device's adaptability to various user needs, although concerns about its weight and portability were raised. Some adjustments to the damping mechanism were suggested for smoother movement. Performance analysis confirmed the device's ability to provide consistent assistance and stability during use. Overall, the HAD showed strong potential to enhance handwriting for children with motor impairments, with recommendations for design refinement to improve comfort and portability.

### 5.3 Results: objective 3

The first five participants (children) were able to hold a pen but had difficulty writing words or drawing different forms on their own. The children had relatively fair gross motor control but they had difficulty holding the pen straight. The damping was thus not necessary, but the HAD helped them to hold the pen straight up. They generally used the sphere handle. They were able to use the HAD and it helped them to draw the required forms. From the occupational therapists' point of view, the lines were better defined and some children were even able to trace the first letter of their name, which they could not do without the device.

**Figure 8:**
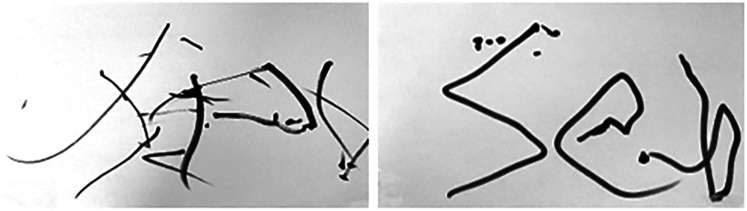
Handwriting of the word “Seb” without the HAD (left) and with the HAD (right).

**Figure 9:**
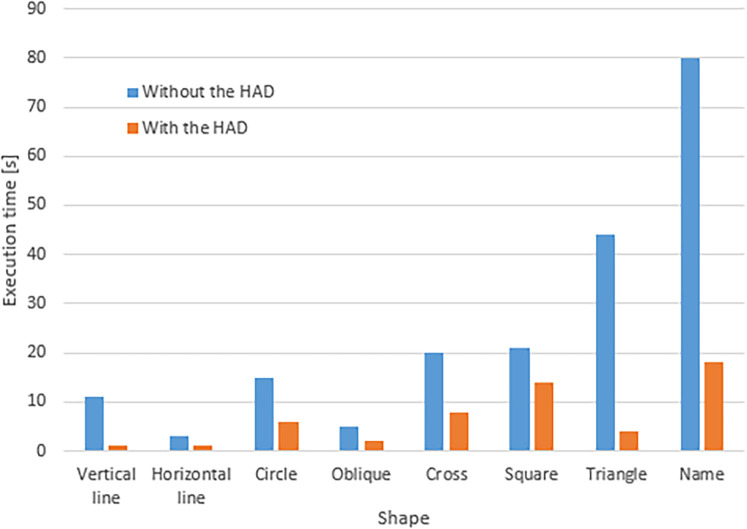
Execution time for different forms with and without the HAD for one adult living with cerebral palsy.

The last participant (an adult with cerebral palsy) had difficulty drawing or writing on his own without the HAD. He used the HAD with angular dampers and a T-shaped handle. The damping helped to stabilize his movements. He was able to draw all the required forms. He was also capable of writing down his name (“Seb”) even though he had never learned to write. All the drawings and writing were performed faster and with more fluidity while using the HAD. Figure 8 shows the difference in the writing of the user with and without the HAD. A qualitative analysis shows that the writing is more legible when the HAD is used. The execution times for the different forms are presented in Figure 9. He mentioned he would have liked to use such an HAD when he was young, so he could have learned how to write on his own.

Figure 9 shows that all the shapes were executed faster with the HAD. In general, the execution with the HAD was 4.81 times faster than without the HAD.

## 6 Discussion

The objective of this paper was to develop a HAD to help children living with movement disorders write and draw. This was done in three parts: (1) establish the current situation amongst the target population, (2) iteratively design a prototype, and (3) perform a preliminary evaluation of the prototype to assess its performance and to guide the development of future iterations. The preliminary evaluation of the HAD conducted in this paper indicates that it facilitates users with physical limitations in writing and drawing, showing promise for those with motor difficulties. Testing with five children and one adult with cerebral palsy demonstrated that HAD provided vertical pen support and improved shape definition, with the adult user achieving faster and more controlled results than with a regular pen.

The objective of the proposed paper aligns with existing literature on the need for assistive technologies (AT) to support individuals with movement disorders in performing daily tasks such as writing and drawing. The work of Lemire et al. proposed a device that is designed to be fixed on a table (18). A pen is attached to the device using a pen holder, which maintains the pen in a fixed orientation. The user interacts with the device using a handle while mechanical dampers and inertia contribute to the stabilization of the user's movements. Hence this device is designed to address key challenges faced by children with movement disorders, particularly those with limited control over their hand movements, by providing mechanical support that enhances both functionality and usability. Therefore the proposed device by Lemire et al. reflects the iterative design and evaluation process that our study advocates, making it a practical and contextually relevant solution for our target population (18).

Moreover the literature indicates that rehabilitation is crucial in the recovery process for movement disorders, such as dystonia. Chiaramonte & Vecchio revealed that implementing an appropriate rehabilitation protocol can enhance motor performance and improve the quality of life, particularly for individuals like musicians who rely heavily on fine motor skills (19). Additionally the study by Carpe et al. emphasizes the role of occupational therapists in providing writing aids and reveals both the positive impact (such as increased confidence, autonomy, and productivity) and the potential challenges associated with these technologies (20). By exploring children's perspectives on writing aids, the study conducted by Carpe et al. provides insights into the enablers and barriers of assistive technology use, informing our approach to designing a handwriting assistive device (HAD) that aligns with the needs and preferences of children with movement disorders. Hence these findings support our research by highlighting the importance of assistive technology for children with physical disabilities in enhancing their engagement in daily activities.

## 7 Conclusion

This study successfully developed a mechanical Handwriting Assistance Device (HAD) that effectively supports individuals with movement disorders in writing and drawing tasks. The preliminary evaluation demonstrated that the HAD enhances pen orientation and control, enabling children with motor difficulties and adults with cerebral palsy to achieve more accurate and faster handwriting compared to using a regular pen. These findings highlight the HAD's potential to improve functional independence and quality of life for users with motor impairments.

Looking ahead, future research should focus on refining the device's ergonomic design and transitioning to an electronic version with adjustable damping capabilities to better accommodate varying user needs. An electronic version could enable real-time damping adjustments, allowing free voluntary movement while dampening involuntary movements, and supporting a gradual reduction in assistance as the child progresses. Additionally, conducting extensive clinical trials with a larger and more diverse population will be essential to validate the device's efficacy and versatility. Exploring avenues for commercialization could also facilitate broader access to this assistive technology, addressing a critical gap in support for individuals with movement disorders. By advancing these areas, the HAD can become a widely available tool that significantly enhances the handwriting capabilities and overall daily functioning of its users.

## Data Availability

The original contributions presented in the study are included in the article/Supplementary Material, further inquiries can be directed to the corresponding authors.

## Footnotes

^1^Penagain. (n.d.) Retrieved Oct. 2018, from http://penagain.net/.

^2^Readi Steadi. (n.d.) Retrieved Oct. 2018, from https://www.readi-steadi.com/.
